# Diversity and Ecology of Fungi from Underexplored and Extreme Environments

**DOI:** 10.3390/jof11050343

**Published:** 2025-04-28

**Authors:** Daniela Isola, Francesc X. Prenafeta-Boldú

**Affiliations:** 1 Department of Agriculture and Forest Sciences (DAFNE), University of Tuscia, Via S. Camillo de Lellis, 01100 Viterbo, Italy; 2 Program of Sustainability in Biosystems, Institute of Agrifood Research and Technology (IRTA), 08140 Caldes de Montbui, Spain

## 1. Introduction

Fungi represent one of the most diverse and ecologically important groups of organisms on Earth, yet much of their biodiversity remains unknown and unexplored [1]. Fungi do not only inhabit the obvious niches that we associate with soil and decaying matter but also thrive in some of the most challenging environments on the planet [2]. Their prolonged evolutionary history has equipped them with strategies and adaptations that allow them to colonize ecological frontiers where few other organisms survive. These environments may result from anthropogenic pressures such as pollution or habitat degradation or may be naturally extreme due to arid, saline, cold, or nutrient-deprived conditions. As a result, extremotolerant and extremophilic fungi represent an emerging research field in mycology [3], both for their relevance to applied sciences and for the novelty of their taxonomic and functional diversity.

This Special Issue presents a collection of nine research articles and one review contributing to the growing field of extremotolerant and extremophilic fungi. The purpose of this Editorial is not only to briefly summarize the compiled contributions but also to provide a broader interpretive context, one that references past research and anticipates directions for future research.

## 2. Overview of Published Articles

A first group of contributions focuses on fungi isolated from anthropogenically altered environments and engineered biosystems. The resilience of fungal communities in the face of induced abiotic stressors is exemplified in the work by Wang et al., who investigate dark septate endophytes and their symbiotic interactions with *Astragalus membranaceus*—a perennial herb used in traditional Chinese medicine—under drought and cadmium stress. Their findings underscore the potential of fungal endophytes in enhancing plant resistance in degraded or contaminated soils. Further in the realm of bioremediation and melanized fungi, Medina-Armijo and coworkers asses the metallotolerance and biosorption capacity of a collection of black fungi exposed to arsenate and chromate, providing promising leads for fungal applications in detoxifying environments polluted with heavy metals and metalloids.

Interestingly, the conditions under which some of those melanized fungal species were repeatedly isolated—particularly in association with plant roots [4]—suggest a possible overlap between the ecological roles of black fungi/black yeasts and dark septate endophytes (DSEs). While the resilience and stress tolerance of melanized fungi make them valuable in biotechnology and plant symbiosis, they also raise concerns regarding their potential to threaten subterranean cultural heritage [4,5]. Moreover, DSEs and human-pathogenic black fungi share key evolutionary and physiological traits, raising intriguing questions about the continuum between plant mutualism/commensalism and opportunism in melanized fungal lineages [6]. These findings point toward an emerging convergence between very distinct research areas. The ecological and industrial significance of industrial fungi is addressed in the work by Lappe-Oliveras et al., who characterize the genetic and phenotypic variability of *Kluyveromyces marxianus* from traditional Mexican agave-based beverages, highlighting the evolutionary and functional traits of yeasts adapted to specific ethanol-producing fermentation processes. Certain fungi can also thrive in completely anaerobic fermentation systems, such as methanogenic bioreactors. Obi and colleagues unveil a surprisingly diverse fungal community that coexists with bacterial consortia, pointing to previously unrecognized roles of fungi in anaerobic ecosystems. These findings are particularly relevant given the current growth and industrial scaling of the biomethane sector, where understanding the full complexity of microbial consortia could enhance process efficiency and stability.

A second group of studies explore fungal communities thriving in natural ecological niches often considered inhospitable because of salinity and aridity. Guerra-Mateo et al. survey the Ascomycete diversity in Western Mediterranean marine sediments, employing direct plating and flocculation techniques to isolate new taxa. The study by Barnés-Guirado and co-authors adds to this narrative by introducing a new genus—*Dactyliodendromyces*—from a hypersaline lagoon, contributing to the taxonomic refinement of the Microascaceae family. This Special Issue also sheds light on saxicolous fungi, with Sastoque et al. describing several novel species from rock-inhabiting fungal communities in Tarragona Province, Spain. Similarly, Gross and colleagues investigate xerophilic Aspergillaceae in the mound nests of *Formica obscuripes*, highlighting how fungal diversity in these microhabitats is shaped by aridity and suggesting potential ecological functions within insect–fungus interactions. At the opposite end in terms of natural extremes, two geographically remote studies extend the scope of this Special Issue to polar ecosystems. Marchetta and colleagues use metabarcoding to profile microfungal communities in Arctic and Antarctic lakes, revealing a remarkable diversity and the presence of cryptic or uncultured lineages, offering insights into the ecological functioning of fungi in cold aquatic environments. This Special Issue concludes with a comprehensive review by Rajakaruna et al. that explores the pharmaceutical and ecological potential of seagrass-associated endophytic and epiphytic fungi, providing a critical synthesis of current knowledge and future research directions in marine mycology.

## 3. Outlook and Prospects

It is important to acknowledge that despite substantial progress, our fundamental understanding of fungal species remains incomplete. New species continue to be isolated and described at a steady pace, yet a considerable knowledge gap on fungal biodiversity persists. Not all fungi can be readily cultured, and standard isolation methods often favor fast-growing or competitive species, while ecologically specialized groups may require prolonged incubation periods or tailored techniques [7]. This highlights the continued relevance of traditional microbiology, even in an era dominated by high-throughput approaches. Culture collections thus remain indispensable—not only as repositories for biotechnologically promising strains but also as foundational resources for calibrating and interpreting large-scale molecular datasets [8]. These collections serve as critical bridges between classical taxonomy and modern omics-driven investigations, enabling a more holistic and accurate understanding of fungal diversity.

Looking ahead, the research on extremophilic and extremotolerant fungi is poised to develop in several promising directions. Advances in environmental sequencing, single-cell genomics, and metabolomics will continue to uncover cryptic diversity and functional potential in fungal communities from poorly explored habitats, from deep ecosystems to the upper limits of the biosphere, and the colonization of artificial or contaminated substrates. Understanding the molecular and physiological mechanisms that underpin fungal survival under different types of stressors, besides those already covered here—e.g., high pressure, ionizing radiation, extreme pH values—will not only illuminate evolutionary strategies but also inform biomimetic and biotechnological innovations.

At the same time, interdisciplinary approaches that integrate ecology, phylogenetics, and materials science are expected to grow in relevance, particularly in the context of climate change, bioremediation, and sustainable resource development. Greater emphasis on symbiotic interactions—whether with plants, insects, bacteria, or other fungi—may also reveal how extremophilic fungi contribute to ecosystem resilience and co-adaptation [9]. Importantly, as the interest in applications using extremotolerant and extremophilic fungi expands across fields such as synthetic biology, medical mycology, pharmacology, the preservation of cultural heritage, and symbiosis-driven agriculture, it is crucial for research to remain grounded in ecological knowledge. Taxonomic accuracy will also be essential in translating extremophile biology into efficient and safe solutions. We hope that this Special Issue will stimulate further research into the untapped potential of fungi from underexplored and extreme environments and foster interdisciplinary dialog across the fields of mycology, ecology, environmental sciences, and biotechnology.
